# Risk factors for postoperative pulmonary complications in elderly patients receiving elective colorectal surgery: A retrospective study

**DOI:** 10.3389/fonc.2022.1002025

**Published:** 2022-09-20

**Authors:** Yuanqiang Dai, Guolin Sun, Hongli Hu, Chun Wang, Hengyue Wang, Yanping Zha, Ying Sheng, Jiong Hou, Jinjun Bian, Lulong Bo

**Affiliations:** Faculty of Anesthesiology, Changhai Hospital, Naval Medical University, Shanghai, China

**Keywords:** colorectal surgery, advanced age, retrospective analysis, risk factors, postoperative pulmonary complications

## Abstract

**Study objective:**

Postoperative pulmonary complications (PPCs) are common and associated with adverse outcomes impairing long-term survival and quality of recovery. This single-centered retrospective study aimed to examine factors associated with PPCs in patients receiving elective colorectal surgery aged ≥60 years.

**Methods:**

Between January 2019 and December 2019, 638 patients at the Shanghai Changhai Hospital who had received elective surgery for colorectal cancer were enrolled in this study. Patients were divided into the PPC group (n=38) and non-PPC group (n=600). Neutrophil–lymphocyte ratio (NLR), platelet–lymphocyte ratio (PLR), red blood cell distribution width (RDW), and systemic inflammatory index (SII) were selected and caculated to indicate preoperative and postoperative inflammatory status. Receiver operating characteristic curve and bivariate correlation analyses were performed to evaluate the identified risk factors.

**Main results:**

The overall incidence of PPCs was approximately 5.96%. Multivariate regression analysis identified age (OR = 1.094, 95%CI 1.038–1.153, *P* = 0.001), preoperative RDW (OR = 1.159, 95%CI 1.025–1.309, *P* = 0.018), and preoperative SII (OR = 1.001, 95%CI 1.000–1.003, *P* = 0.035) as independent risk factors for PPCs. The cut-off values of age, preoperative RDW, and preoperative SII for predicting PPCs were 69.5 (sensitivity 0.658, specificity 0.653), 13.2 (sensitivity 0.789, specificity 0.552) and 556.1 (sensitivity 0.579, specificity 0.672), respectively.

**Conclusions:**

Age, preoperative RDW, and preoperative SII were identified as independent risk factors for PPC occurrence in elderly patients receiving elective colorectal surgery. Further studies are warranted to evaluate whether normalization of preoperative RDW and SII, as modifiable risk factors, are associated with improved surgical outcomes.

## Introduction

Colorectal cancer ranks third in terms of global cancer incidence and is the second leading cause of cancer-related mortality according to the Global Cancer Statistics 2020 (1). Surgery is the primary curative treatment for colorectal cancer. Perioperative complications after major surgery remain a considerable healthcare burden and are associated with increased mortality and morbidity (2). Postoperative pulmonary complications (PPCs) are common, with an incidence of 2% to 40%, and are associated with adverse outcomes impairing long-term survival and quality of recovery (3). Several studies have been performed to explore and determine the perioperative risk factors for PPCs (4).

Red blood cell distribution width (RDW) is a simple measure of the broadness of erythrocyte size distribution, conventionally called anisocytosis. Increases in RDW observed in patients are generally associated with chronic inflammation or poor nutritional status, and RDW has been suggested as a long-term inflammatory biomarker (5). A recent retrospective study involving 21,842 patients receiving non-cardiac surgery indicated that increased preoperative RDW is associated with increased long-term mortality (6). The systemic immune-inflammation index (SII) is a derivative and new inflammatory biomarker, derived from neutrophils (NEUT), lymphocytes, and platelet counts, and has been used to evaluate the outcome of patients with solid cancers and coronary heart disease (7). Our recent prospective study, with a total of 76 patients aged >65 years receiving elective orthopedic surgery, indicated that postoperative cognitive decline (POCD) in such patients was associated with a significantly high level of SII admission (8). SII was independently associated with the occurrence of POCD in the study cohort. However, studies that investigated the relationship between routine blood test results and PPCs are few.

This single-centered study aimed to examine factors associated with PPCs in patients with colorectal cancer aged ≥60 years. We acknowledge that factors associated with PPCs after colorectal surgery procedures are multifactorial. The current study was conducted to elucidate the relationship of routine blood test results with PPCs.

## Materials and methods

### Study design and data source

Ethical approval for this retrospective study was provided by the Ethics Committee of Changhai Hospital (CHE 2020-148), and the requirement for obtaining informed consent was waived. This study adhered to the ethical standards set by the National Health Commission of the People’s Republic of China.

DoCare Anesthesia Clinical Information System (Medical System,V3.1.0 Build153; Suzhou, China) and electronic case system of Shanghai Changhai Hospital were used for study data retrieval. Patients were systematically identified with the keywords “colorectal cancer”, “radical resection”, and “general anesthesia”. Patients who received radical surgery for colorectal cancer for the first time from January 2019 to December 2019 were selected, and patients aged >60 years with American Society of Anesthesiologists (ASA) physical status I–III, and complete clinical data were screened and selected for this retrospective analysis. Patients who met any of the following criteria were excluded: serious intraoperativecardiovascular and cerebrovascular events (such as cardiac arrest, acute myocardial infarction, and acute cerebral infarction); patients with preoperative severe respiratory diseases (such as severe asthma, an acute exacerbation of chronic obstructive pulmonary disease, history of pulmonary tissue resection leading to significant loss of pulmonary function, pulmonary hypertension with any cause, and respiratory insufficiency or failure); patients with preoperatively existing tumor metastasis or receiving long-term chemotherapy; lack of clinical data related to the study; general anesthesia without endotracheal intubation; and no radical surgical treatment was performed.

### Data collection

For eligible patients in this study, the following relevant information was retrieved: demographic data (sex, age, height, weight, and smoking status; history of hypertension, diabetes, coronary heart disease, stroke; pulmonary imaging changes; pulmonary underlying diseases; and immune system diseases); surgical information (surgical site: rectum, use of laparoscopy, enterostomy, intestinal adhesion, combined viscerectomy, anesthesia time, operation time, blood loss, urine volume, crystal volume, colloid volume, red blood cell suspension, plasma, and perioperative sufentanil usage); results of preoperative and postoperative blood tests (white blood cell count, neutrophil count, neutrophil percentage, hemoglobin (HGB), albumin (ALB), RDW, platelet count, monocyte count, and lymphocyte count); and prognosis during hospitalization (PPC, recovery and discharge, death, and total length of stay).

Neutrophil–lymphocyte ratio (NLR), platelet–lymphocyte ratio (PLR), and SII were selected and calculated to indicate preoperative and postoperative inflammatory status. NLR is defined as the ratio of neutrophil count to lymphocyte count; PLR is defined as the ratio of platelet count to lymphocyte count; and SII is defined as the ratio of neutrophil count multiplied by platelet count to lymphocyte count (9).

We also compared and analyzed the changes in routine blood test results, including NLRs, PLRs, and SII, preoperatively and postoperatively in both groups. Among them, HGB, ALB, platelet count, and lymphocyte count demonstrated a downward trend postoperatively; thus, the preoperative value minus the postoperative value was considered the change value (Δ) for comparison; the other remaining indicators were compared with the postoperative value minus the preoperative value.

### PPCs

PPCs are defined as new-onset events of respiratory complications during postoperative hospitalization, mainly including pulmonary infection, atelectasis, pleural effusion, and acute respiratory failure. PPC was measured according to the Melbourne Group Scale (MGS) scoring criteria. The MGS scoring criteria included: body temperature > 38 °C; white blood cell count increased to >11.2×10^9^/L; postoperative atelectasis or chest X-ray findings; new cough or/and purulent sputum; positive sputum pathogen culture; postoperative clinical diagnosis of new pneumonia; blood oxygen saturation <90% when breathing air; and prolonged hospitalization. PPC was diagnosed when the patient meets four or more of the criteria.

### Statistical analysis and data management

Normality of continuous data was tested by the Kolmogorov–Smirnov method. According to data distribution, variance analysis and post-hoc verification were used for continuous variables conforming to normal distribution, and the results are expressed as mean ± standard deviation (X ± S). The Mann–Whitney U test was performed on non-normally distributed data, and the results are presented as median and quartile spacing M (Q25, Q75). The chi-square test was used to assess differences between groups, and the results are expressed as number of cases or percentage (%). Multivariate logistic regression analyses were performed incorporating all factors with *P* < 0.1 on bivariate analyses and a prevalence of at least 1%, as well as other variables with potentially clinical importance, using the backward stepwise selection technique and accepting statistical significance at *P* < 0.05. *A* bivariate correlation analysis was performed to verify the linear relationship of PPCs with diagnostic conditions and associated risk factors in order to clarify the positive and negative correlations between variables. PPCs, dyspnea, pneumonia, pleural effusion, atelectasis, acute respiratory failure, T > 38 °C within 7 days postoperatively, SpO_2_< 90%, new cough sputum were considered “variables”. The test of significance option was set to “two-tailed test”. Correlation coefficients were set to the “Pearson” option. In the calculation results, a negative value of the correlation coefficient indicates a negative correlation between the two variables, and a positive value indicates a positive correlation between the two variables. Results are expressed with OR values and 95%CI. *P* values < 0.05 were considered significant. All analyses were performed using IBM SPSS^®^ Statistics V22 (IBM Corporation, NY, USA).

## Results

### General characteristics and postoperative outcomes

**Figure 1:**
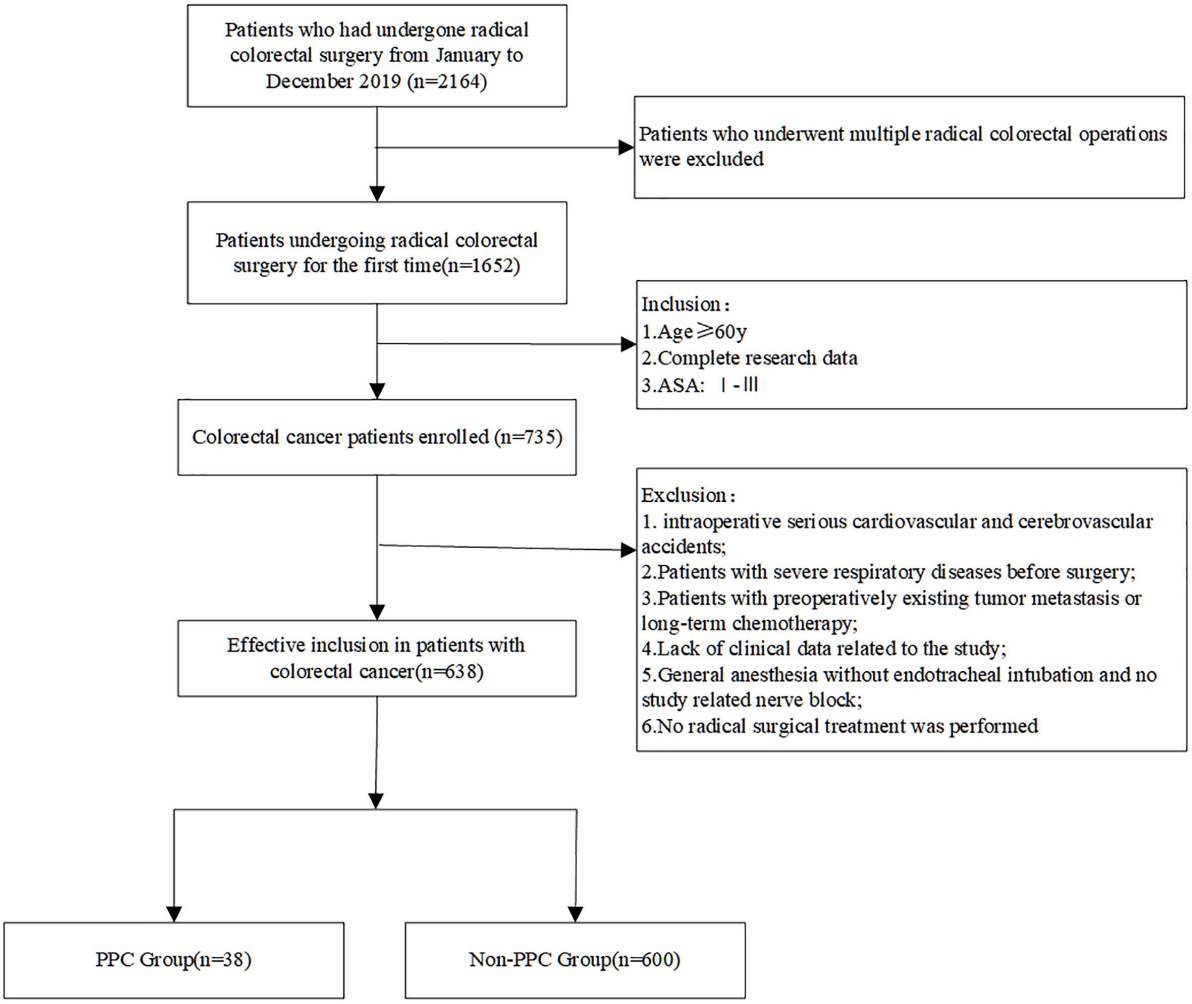
Flow diagram of patient inclusion.

In total, 2,164 patients with colorectal cancer received surgery during the study period, including 1,652 patients who underwent radical resection for the first time (**Figure 1**). Of the patients, 735 patients aged >60 years with complete data were selected. After applying the exclusion criteria, 638 patients were finally included in the study. The mean age of the patients was 68.5 ± 6.0 years, and 65.5% (n = 418) of the patients were men. The 30-day all-cause mortality was approximately 0.47% (3/638) (**Figure 1**; **Table 1**). According to the presence or absence of PPCs postoperatively, the patients were divided into two groups: the PPC group (n = 38) and non-PPC group (n = 600). The overall incidence of PPCs was approximately 5.96%. The characteristics and postoperative in-hospital outcomes of patients are presented in **Table 1**. The mean age was higher in the PPC group (72.8 ± 7.4 vs. 68.3 ± 5.8., *P* < 0.001) than that in the non-PPC group. The median length of hospital stay of the PPC group was significantly longer than that of the non-PPC group (13.7 ± 5.9 vs. 11.3 ± 3.9, *P* < 0.001).

**Table 1:** General characteristics and postoperative outcomes of patients.

Parameter	PPCs (n = 38)	Non-PPCs (n = 600)	*P*
Sex, n (male%)	26(68.4)	392 (65.3)	0.698
Age, y	72.8 ± 7.4	68.3 ± 5.8	0.000^*^
BMI, kg/m2	22.7 ± 3.6	23.1 ± 3.3	0.407
ASA, n			0.786
I, n	15(39.5)	247(41.2)	
II, n	16(42.1)	267(44.5)	
III, n	7(18.4)	86(14.3)	
Smoking history, n (%)	7(18.4)	132(22.0)	0.604
Hypertension, n (%)	18(47.4)	255(42.5)	0.556
Diabetes mellitus, n (%)	6(15.8)	85(14.2)	0.781
Coronary heart disease, n (%)	3(7.9)	31(5.2)	0.468
Stroke, n (%)	0(0.0)	18(3.0)	0.279
Perioperative changes in lung CT or X-ray, n (%)	22(57.9)	287(47.8)	0.229
Non-acute (severe) pulmonary disease, n (%)	1(2.6)	30(5.0)	0.510
Autoimmune diseases, n (%)	0(0.0)	4(0.7)	0.614
Hospital deaths, n (%)	2(5.26)	1(0.01)	0.000^*^
Length of hospital stay, days	13.7 ± 5.9	11.3 ± 3.9	0.000^*^

PPCs, postoperative pulmonary complications; BMI, body mass index; ASA, American Society of Anesthesiologists; CT, computed tomography.

*P value < 0.05, with statistical significance.

### Risk factors for PPCs

In order to investigate the risk factors for PPCs in elderly patients receiving elective colorectal surgery, surgical and anesthesia characteristics of the patients were compared (**Table 2**). Interestingly, no significant differences in tumor surgical site, use of laparoscopy, enterostomy, or combined visceral resection were identified between the groups. The operation time and duration of anesthesia were also comparable between the groups. The amount of opioids used perioperatively was generally considered a risk factor for PPCs; however, no significant differences in intraoperative and postoperative use of sufentanil were observed between the groups (**Table 2**). Additionally, we examined whether perioperative fluid and blood managements were associated with PPC occurrence in the patients. As presented in **Table 3**, no significant differences in the amount of urine output, fluid infusion, or the ratio of colloid liquid to crystal liquid were observed between the two groups; however, the amount of intraoperative blood loss in the PPC group was higher than that in the non-PPC group (*P* = 0.044). Meanwhile, a significant increase in the proportion of patients receiving blood transfusion was also noted in the PPC group (21.1% vs 7.5%, *P* = 0.003) (**Table 3**).

**Table 2:** Surgical and anesthesia characteristics of patients.

Variable	PPCs(n = 38)	Non-PPCs(n = 600)	*P*
Surgical site			0.070
Rectum	16(42.1)	343(57.2)	
Colon	22(57.9)	257(42.8)	
Laparoscopy assistance, n (%)	14(36.8)	264(44.0)	0.388
Enterostomy performed, n (%)	8(21.1)	196(32.7)	0.137
Adhesiolysis performed, n (%)	8(21.1)	91(15.2)	0.331
Combined viscerectomy, n (%)	3(7.9)	82(13.7)	0.310
Anesthesia time, hour	3.9 ± 1.6	3.7 ± 1.5	0.498
Operation time, hour	3.3 ± 1.6	3.1 ± 1.5	0.315
Intraoperative sufentanil use, μg	43.0 ± 10.0	45.3 ± 10.9	0.206
postoperative sufentanil use, μg	55(0,80)	58(0,80)	0.651
PCA with sufentanil, n(%)	27(71.1)	391(65.2)	0.459

PPCs, postoperative pulmonary complications; PCA, patient-controlled analgesia.

**Table 3:** Perioperative fluid and blood management in patients.

Variable	PPCs(n = 38)	Non-PPCs(n = 600)	*P*
Blood loss, L	0.2(0.1,0.3)	0.2(0.1,0.2)	0.044^*^
Urine output, L	0.6(0.4,1.0)	0.6(0.5,1.0)	0.600
Crystal liquid infusion, L	1.7(1.5,2.1)	1.8(1.3,2.2)	0.740
Colloid liquid infusion, L	0.5(0.5,1.0)	0.5(0.5,1.0)	0.168
Ratio of colloid liquid to crystal liquid	0.3(0.2,0.6)	0.3(0.2,0.5)	0.244
Total liquid infusion volume, L	2.4(2.1,2.8)	2.3(2.1,2.8)	0.776
Patients with intraoperative blood transfusion, n (%)	8(21.1)	45(7.5)	0.003^*^

*P value < 0.05, with statistical significance.

Considering that this retrospective study aimed to explore risk factors for PPCs based on results of perioperative blood tests, we then analyzed relevant variables obtained or calculated. **Table 4** presents the blood test results between the groups preoperatively and postoperatively. The PPC group had significantly lower preoperative plasma HGB (P = 0.001) and ALB (P < 0.001) levels but significantly higher preoperative RDW (*P* < 0.001) and NEUT (*P*=0.021) compared with those in the non-PPC group. In terms of inflammation related indices, the NLRs (*P* = 0.022), PLRs (*P* = 0.014), and SII (*P* = 0.007) in the PPC group were significantly higher than those in the non-PPC group. Postoperatively, the PPC group had significantly higher blood NEUT (*P* = 0.029) and RDW (*P* < 0.001) but significantly lower HGB (P = 0.018) and ALB (P = 0.003) levels than the non-PPC group. However, when comparing the inflammatory related indices, only the patients in the PPC group had a significantly higher SII (*P* = 0.042) (**Table 4**). The changes in the routine blood test results preoperatively and postoperatively in both groups were compared, and only the change in platelet count was significantly different between both groups (*P* = 0.009) (**Table 5**).

**Table 4:** Results of preoperative and postoperative blood tests in patients.

Variable	PPCs (n=38)	Non-PPCs (n=600)	*P*
**Preoperative**			
WBC, 10^9^/L	6.0(5.2,7.9)	5.8(4.8,6.8)	0.197
NEUT, 10^9^/L	3.8(3.0,4.9)	3.4(2.7,4.3)	0.021^*^
GRA,%	61.0 ± 9.1	59.2 ± 9.6	0.254
HGB, g/L	115.5 ± 25.8	127.4 ± 21.8	0.001^*^
ALB, g/L	37.8 ± 5.1	40.5 ± 3.9	0.000^*^
RDW, %	13.9(13.2,17.1)	13.1(12.4,14.0)	0.000^*^
PLT, 10^9^/L	244.7 ± 90.1	221.1 ± 71.1	0.052
MONO, 10^9^/L	0.5(0.4,0.7)	0.5(0.4,0.6)	0.175
LY, 10^9^/L	1.6(1.1,2.1)	1.6(1.3,2.0)	0.429
NLRs	2.5(1.7,3.7)	2.1(1.6,2.8)	0.022^*^
PLRs	168.7(117.4,206.2)	131.6(102.9,172.8)	0.014^*^
SII	602.5(347.4,932.0)	420.4(316.8,645.0)	0.007^*^
**Postoperative**			
WBC, 10^9^/L	10.7 ± 3.1	10.2 ± 3.4	0.454
NEUT, 10^9^/L	9.5 ± 3.4	8.3 ± 3.1	0.029^*^
GRA, %	82.7 ± 5.9	80.7 ± 7.9	0.131
HGB, g/L	108.5 ± 18.3	115.9 ± 18.8	0.018^*^
ALB, g/L	32.3 ± 4.6	34.3 ± 3.8	0.003^*^
RDW, %	14.4(13.0,17.2)	13.0(12.4,14.1)	0.000^*^
PLT, 10^9^/L	208.5 ± 65.1	199.7 ± 64.7	0.413
MONO, 10^9^/L	0.8(0.6,1.0)	0.8(0.6,1.0)	0.659
LY, 10^9^/L	0.8(0.7,1.1)	0.9(0.6,1.2)	0.500
NLRs	10.1(7.4,15.9)	8.9(6.1,13.4)	0.064
PLRs	234.1(187.4,324.2)	225.0(160.7,308.5)	0.278
SII	2023.9(1457.4,3522.2)	1734.1(1099.4,2717.7)	0.042^*^

WBC, white blood cell count; NEUT, neutrophils count; GRA, neutrophilic granulocyte percentage; HGB, hemoglobin; ALB, albumin; RDW, red blood cell distribution width; PLT, platelet count; MONO, monocyte count; LY, lymphocyte count; NLRs, neutrophil to lymphocyte ratios; PLRs, platelet to lymphocyte ratios; SII, systemic immune-inflammation index.

*P value < 0.05, with statistical significance.

**Table 5:** Value changes in blood test results preoperatively and postoperatively.

Variable	PPCs (n = 38)	Non-PPCs (n = 600)	*P*
Δ WBC, 10^9^/L	4.2 ± 3.5	4.2 ± 3.3	0.901
Δ NEUT, 10^9^/L	5.2 ± 3.5	4.7 ± 3.1	0.379
Δ GRA,%	21.7 ± 9.4	21.5 ± 10.2	0.931
Δ HGB, g/L	7.1 ± 21.7	11.5 ± 15.6	0.095
Δ ALB, g/L	5.5 ± 5.8	6.2 ± 4.7	0.379
Δ RDW, %	0.1 ± 0.6	0.0 ± 0.7	0.331
Δ PLT, 10^9^/L	36.2 ± 58.6	21.4 ± 31.3	0.009*
Δ MONO, 10^9^/L	0.3(0.1,0.5)	0.3(0.1,0.5)	0.931
Δ LY, 10^9^/L	0.7(0.3,1.0)	0.7(0.4,1.1)	0.660
Δ NLRs	8.3(4.7,12.6)	6.6(4.0,11.0)	0.254
Δ PLRs	73.2(38.5,134.1)	81.6(33.9,154.4)	0.548
Δ SII	1330.2(778.2,2496.2)	1244.7(689.1,2126.1)	0.397

Δ, difference between preoperative and postoperative blood indices; WBC, white blood cell count; NEUT, neutrophil count; GRA, neutrophilic granulocyte percentage; HGB, hemoglobin; ALB, albumin; RDW, red blood cell distribution width; PLT, platelet count; MONO, monocyte count; LY, lymphocyte count; NLRs, neutrophil to lymphocyte ratios; PLRs, platelet to lymphocyte ratios; SII, systemic immune-inflammation index.

*P value < 0.05, with statistical significance.

Variables with significant differences were then selected for the univariate logistic regression analysis, and age, preoperative NEUT, preoperative HGB, preoperative ALB, preoperative RDW, preoperative NLRs, preoperative PLRs, preoperative SII, and intraoperative blood transfusion were identified as risk factors (**Table 6**). In the subsequent multivariate regression analysis, age (OR = 1.094, 95%CI 1.038–1.153, *P* = 0.001), preoperative RDW (OR = 1.159, 95%CI 1.025–1.309, *P* = 0.018), and preoperative SII (OR = 1.001, 95%CI 1.000–1.003, *P* = 0.035) were identified as independent risk factors for PPCs (**Table 6**).

**Table 6:** Logistics analysis of risk factors related to postoperative pulmonary complications.

Variable	Univariate analysis				Multivariate analysis			
	β value	OR	95%CI	*P* value	β value	OR	95%CI	*P* value
Age	0.104	1.109	1.058-1.163	0.000^*^	0.090	1.095	1.037-1.155	0.001^*^
Preoperative NEUT	0.218	1.243	1.057-1.463	0.008^*^	-0.031	0.970	0.922-1.020	0.234
Preoperative HGB	-0.022	0.979	0.966-0.992	0.002^*^	-0.015	0.985	0.954-1.018	0.381
Preoperative ALB	-0.148	0.862	0.799-0.930	0.000^*^	-0.085	0.919	0.834-1.012	0.087
Preoperative RDW	0.162	1.176	1.078-1.282	0.000^*^	0.174	1.190	1.048-1.351	0.007^*^
Preoperative NLRs	0.176	1.193	1.036-1.373	0.014^*^	-0.034	0.967	0.670-1.395	0.856
Preoperative PLRs	0.004	1.004	1.000-1.008	0.031^*^	-0.005	0.995	0.988-1.002	0.204
Preoperative SII	0.001	1.001	1.000-1.001	0.002^*^	0.002	1.002	1.000-1.003	0.022^*^
Bleeding	1.079	2.941	0.649-13.328	0.162	0.755	2.127	0.367-12.327	0.400
Intraoperative blood transfusion	1.191	3.289	1.424-7.595	0.005^*^	0.325	1.384	0.434-4.417	0.583

NEUT, neutrophil count; HGB, hemoglobin; ALB, albumin; RDW, red blood cell distribution width; NLRs, neutrophil to lymphocyte ratios; PLRs, platelet to lymphocyte ratios; SII, systemic immune-inflammation index.

*P value < 0.05, with statistical significance.

**Figure 2:**
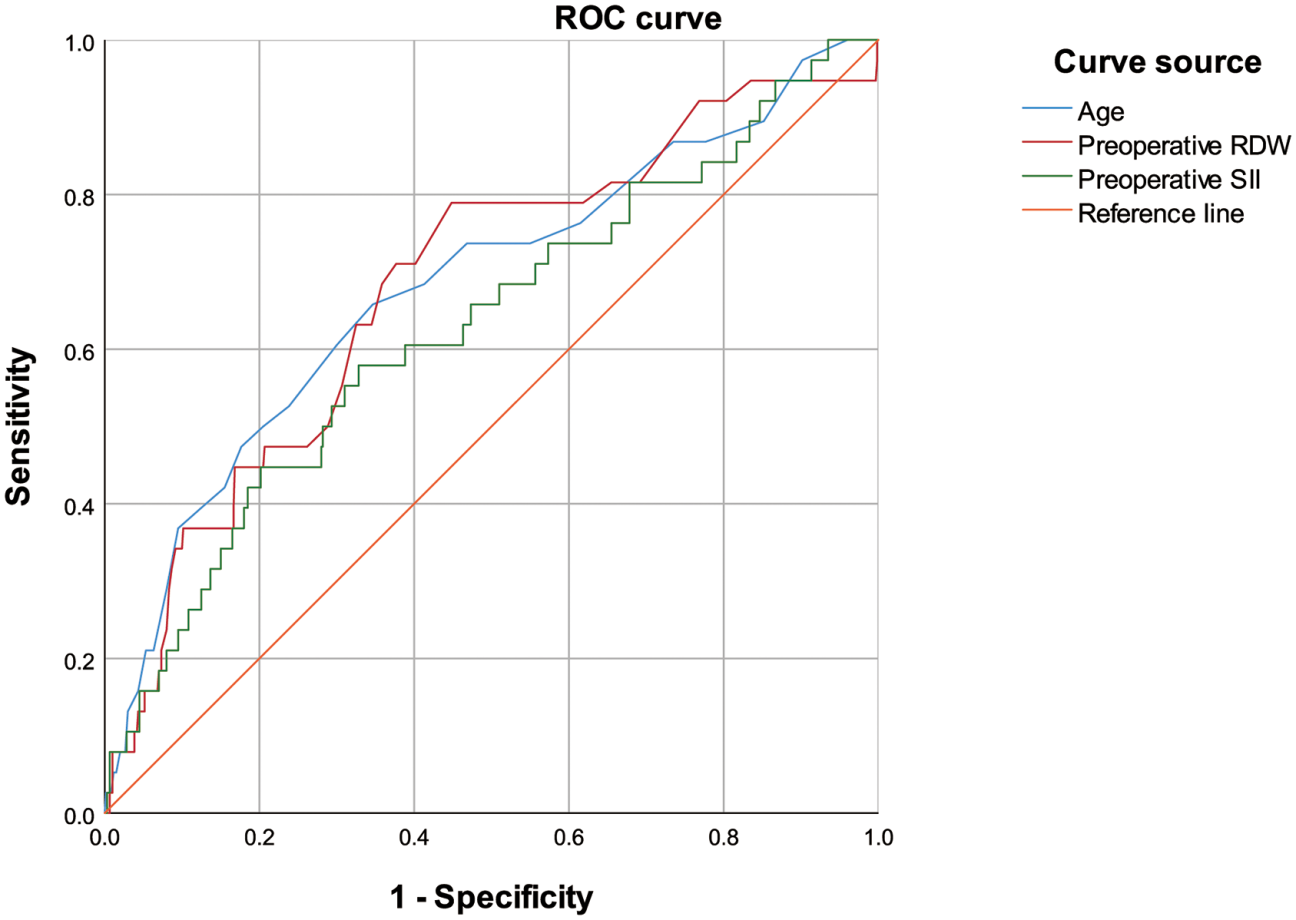
Receiver operating characteristic curve of age, preoperative red blood cell distribution width, and preoperative systemic inflammatory index for predicting postoperative pulmonary complications.

**Figure 3:**
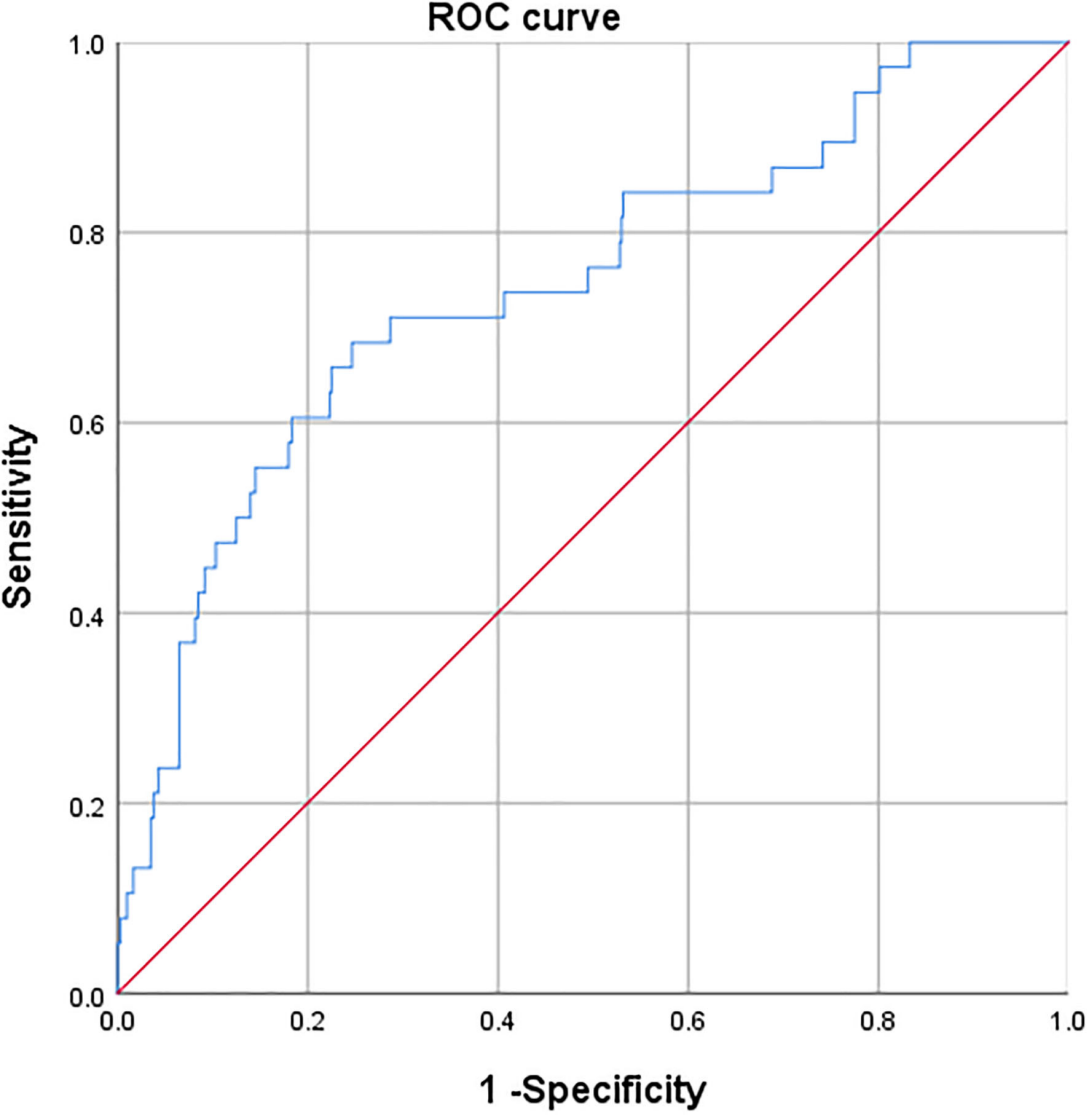
Receiver operating characteristic curve of three independent risk factors for predicting postoperative pulmonary complications.

A receiver operating characteristic curve (ROC) analysis of the risk factors age, preoperative RDW, and preoperative SII, was performed to predict the occurrence of PPCs, with area under the curve of 0.683 (95%CI 0.586–0.779, *P* = 0.000), 0.683 (95%CI 0.590–0.775, *P* = 0.000) and 0.629 (95%CI 0.532–0.727; *P* = 0.007), respectively (**Table 7**; **Figure 2**). The cut-off values of age, preoperative RDW, and preoperative SII for predicting PPCs were 69.5 (sensitivity 0.658, specificity 0.653), 13.2 (sensitivity 0.789, specificity 0.552), and 556.1 (sensitivity 0.579, specificity 0.672), respectively. A risk factor prediction model with the abovementioned three independent risk factors for the occurrence of PPCs was also established, and the ROC curve analysis was performed. The area under the ROC curve was 0.744 (with the sensitivity 0.684, and the specificity 0.753), and the Youden index was 0.437 (**Figure 3**).

**Table 7:** Cut-off value for age, preoperative RDW, and preoperative SII for predicting postoperative pulmonary complications.

Parameter	Cut-off value	Specificity	Sensitivity	AUC	Youden index	95% CI	*P* value
Age	69.5	0.653	0.658	0.683	0.311	0.586-0.779	0.000^*^
Preoperative RDW	13.2	0.552	0.789	0.683	0.341	0.590-0.775	0.000^*^
Preoperative SII	556.1	0.672	0.579	0.629	0.251	0.532-0.727	0.007^*^

RDW, red blood cell distribution width; SII, systemic immune-inflammation index.

*P value < 0.05, with statistical significance.

Finally, a bivariate correlation analysis was performed using the independent risk factors for PPCs and PPCs. The results revealed that age, preoperative RDW, and preoperative SII were positively correlated with PPCs and its subtypes. Additionally, with the increase of values in age, preoperative RDW, and preoperative SII, the probability of occurrence of PPCs demonstrated an upward trend (**Table 8**).

**Table 8:** Bivariate correlation analysis of preoperative related factors and postoperative pulmonary complications.

	Correlation	PPCs	Dyspnea	Pneumonia	Pleural effusion	Atelectasis	Acute respiratory failure	T > 38 °C within 7 days postoperatively	SpO_2_ < 90%	New cough and/or sputum
Age	Correlation Coefficient	0.179	0.131	0.111	0.077	0.034	0.060	-0.020	0.043	0.154
	*P* values	0.000^*^	0.001^*^	0.005^*^	0.052	0.387	0.130	0.609	0.276	0.000^*^
Preoperative RDW	Correlation Coefficient	0.156	0.008	0.035	0.035	0.010	0.052	0.099	0.070	0.155
	*P* values	0.000^*^	0.847	0.382	0.384	0.795	0.191	0.012^*^	0.077	0.000^*^
Preoperative SII	Correlation Coefficient	0.137	0.022	0.087	0.039	0.085	0.201	0.044	0.165	0.051
	*P* values	0.001^*^	0.571	0.027^*^	0.321	0.031^*^	0.000^*^	0.262	0.000^*^	0.198

HGB, hemoglobin; ALB, albumin; RDW, red blood cell distribution width; NLRs, neutrophil to lymphocyte ratios; PLRs, platelet to lymphocyte ratios; SII, systemic immune-inflammation index; PPCs, pulmonary complications.

*P value < 0.05, with statistical significance.

## Discussion

This study demonstrated that age, preoperative RDW, and preoperative SII were significant predictors of PPCs in elderly patients receiving elective colorectal surgery. We also constructed the ROC curve of cut-off values of age, preoperative RDW, and preoperative SII for predicting PPCs. To the best of our knowledge, this is the first study to evaluate the role of perioperative RDW and SII for predicting PPCs in elderly patients receiving elective colorectal surgery.

Surgical trauma and the influence of the tumor itself can produce inflammatory reactions in the body (10). Among all of the inflammatory cells, neutrophils play an important role in the inflammatory processes, while lymphocytes are involved in the regulation of the immune system. In this study, NLR, PLR, and SII were used to analyze the preoperative and postoperative inflammatory states. NLR is the ratio of neutrophil to lymphocyte count, and is considered a hematological marker of systemic inflammation. In the recent years, NLR and PLR have been used as inflammatory indices for inflammation and severity of diseases. It is widely recognized that the systemic inflammatory response accompanies the development of cancer, and thus providing us with new insights and methods for evaluating patients’ systematic inflammation status and outcomes (11). Some studies have reported that NLR can reflect early postoperative complications in order to achieve early diagnosis and treatment (12). NLR is also considered a predictive marker for patients with colorectal cancer, which is of great significance for predicting preoperative metastasis and evaluating postoperative prognosis (13). The association of PLRs with tumor survival and progression has been reported, and a PLR>150 was identified as an independent predictor of tumor recurrence in patients with hepatocellular carcinoma (14, 15). SII is a new inflammatory biomarker derived in recent years, defined as (platelet*neutrophil)/lymphocyte count. Based on the relationship between neutrophils, lymphocytes, and platelet count, SII is often used for the clinical evaluation of different disease states and has demonstrated a substantial predictive effect on the progression and treatment prognosis of patients with tumors (7,^16^). A very recent study, including 548 patients with stage I–II gastric cancer after receiving radical surgery, has suggested that preoperative low SII (<508.3) was associated with a significantly higher 5-year survival rate (17). Meanwhile, our study discovered that an SII value > 556.1 was associated with a higher incidence of PPCs.

In our study, the preoperative NLR, PLR, and SII in the PPC group were significantly higher than those in the non-PPC group, suggesting that these variables have potential for predicting PCC occurrence. Since these variables can be easily obtained by routine blood testing, their roles deserve further evaluation. Perioperative anemia has long been considered a risk factor for perioperative complications in patients receiving surgery. Our study also indicated that the pre- and postoperative anemia, indicated by a low threshold of HGB level, was associated with PPC occurrence. However, HGB was not identified as an independent risk factor for PPC. The underlying reasons may be the relatively low number of PPC occurrences, or that HGB is less sensitive than the RDW.

The observed association of RDW and SII with PPCs deserves further exploration, considering that chronic inflammation might exist preoperatively and that both may be associated with unfavorable outcomes. RDW was again commonly used in the assessment of anemia, while being recently regarded as an indicator of long-term inflammation (18). The release of inflammatory factors, oxidative stress, and poor nutritional status are perceived as potential causes of RDW elevation, leading to retention of abnormally sized red cells (19). Increased values in RDW have been considered a negative predictor of survival in several types of malignancies (20). A recent retrospective study including 591 patients with colorectal cancer has reported that only patients with early-stage colorectal cancer may have a worse survival when presenting with an elevated RDW (6). Due to incomplete data collection, information on the depth of tumor invasion, node involvement metastatic disease, TNM stage, and tumor grading for each patient was not retrieved, and thus were not analyzed for their association with PPCs. A recent retrospective cohort study has reported that RDW values between 14.8% and 15.8%, and >15.8% were associated with increased long-term mortality after noncardiac surgery (21). Our study discovered that an RDW value > 13.2% was associated with a significant increase in PPC occurrence. Studies exploring whether normalization of the RDW benefits perioperative outcomes including PPCs in patients receiving surgery will be of interest. Since RDW is a modifiable variable, whether it can be used for risk prediction or stratification for PPCs deserves further research. Moreover, patients with high RDW and SII values may be more prone to perioperative hemodynamic instability, amplified inflammatory response, and mediated postoperative adverse outcomes including PPCs.

Advanced age is a certain risk factor for perioperative complications of patients receiving abdominal surgery due to a decline in physical function and increased comorbidities (22). Our study also confirmed that age is risk factor for PPCs in this population. Interestingly, limited studies have explored the effect of aging on perioperative inflammatory response. One recent retrospective cohort study, with a total of 25,095 patients who received cardiac surgeries, has reported that age was strongly associated with a reduced prevalence of postoperative systemic inflammatory response syndrome (23). This inverse association indicated that an overall reduced postoperative immune response in aging population, also known as immunosenescence, may influence perioperative medication strategies and deserves further research.

Moreover, the incidence of PPCs in our cohort is relatively lower than that of previous studies by approximately 5% to 33% (24, 25). This might be caused by differences among institutional guidance or experiences. Thus, the results can only represent the correlation of related factors included in this study, rather than absolute causality, and may not apply to other abdominal surgical populations. The sensitivity and specificity of the ROC curves were not particularly excellent, which may be mainly caused by the low incidence of PPCs in our study population. Therefore, the value of preoperative RDW and SII for predicting PPCs deserves further exploration.

This study also had some limitations. Firstly, this was a small-sample single-center retrospective study, and the patients who had received colorectal surgery were aged >60 years, which may have resulted in selection bias and confounding factors. Secondly, we did not examine the percentage of patients receiving blood transfusion preoperatively, which might skew the value of RDW. Thirdly, in order to determine whether the present risk factors have high predictive power, multi-center clinical large samples and observational studies need to be conducted. Lastly, other inflammatory markers, such as hs-CRP, procalcitonin, and hematologic parameters such as mean platelet volume, and immature and fragmented platelet forms were not evaluated in the current study, which deserve further investigation. Pneumoperitoneum could affect the lung mechanics in several ways; thus, the application and duration of intraoperative pneumoperitoneum are considered risk factors for PPCs in patients receiving abdominal surgeries. However, our data revealed that the proportion of patients undergoing laparoscopy assistance for colorectal surgery was comparable between the two groups, and no significant difference in the incidence of PPCs was observed between both groups. Moreover, intraoperative ventilation strategies, management of perioperative use of muscle relaxants, and several reported risk factors for PPCs were not examined thoroughly, and deserve further investigation.

In conclusion, by analyzing the perioperative related factors in elderly patients with colorectal cancer, this study identified age, preoperative RDW, and preoperative SII as independent risk factors for PPC occurrence. Further studies comprehensively evaluating the potential risk factors for colorectal surgery and related PPCs are necessary in the future. Future studies should also clarify whether normalization of preoperative RDW and SII, as modifiable risk factors, may improve surgical outcomes.

## Data availability statement

The original contributions presented in the study are included in the article/Supplementary Material. Further inquiries can be directed to the corresponding authors.

## Ethics statement

The studies involving human participants were reviewed and approved by the Ethics Committee of Changhai Hospital. The ethics committee waived the requirement of written informed consent for participation.

## Author contributions

LB, YD, GS, and JB designed the study. GS, CW, HH, HW, YZ, YS, and JH contributed to the conduct of the study. YD, GS, HH, and HW performed the data analysis. LB and YD were the major contributors to writing the first draft of the manuscript. All authors listed reviewed and approved the final version of the manuscript.

## Funding

This work was supported by the Shanghai Science and Technology Committee Rising-Star Program [grant number 19QA1408500] and the “234 Discipline Construction Climbing Plan” of the Changhai Hospital, Naval Medical University [grant number 2020YXK053].

## Conflict of interest

The authors declare that the research was conducted in the absence of any commercial or financial relationships that could be construed as a potential conflict of interest.

## Publisher’s note

All claims expressed in this article are solely those of the authors and do not necessarily represent those of their affiliated organizations, or those of the publisher, the editors and the reviewers. Any product that may be evaluated in this article, or claim that may be made by its manufacturer, is not guaranteed or endorsed by the publisher.
